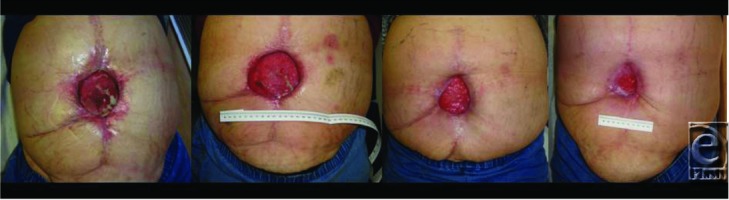# Negative Pressure Wound Therapy for Abdominal Wall Reconstruction

**Published:** 2013-10-31

**Authors:** Kashyap K. Tadisina, Karan Chopra, Jennifer Sabino, John S. Maddox, Sheena Samra, Abhishake Banda, Devinder P. Singh

**Affiliations:** Division of Plastic Surgery, University of Maryland Medical Center, Baltimore, Md

**Keywords:** negative pressure wound therapy, vacuum-assisted closure, chronic wound management, instillation therapy, High-risk wounds

**Figure:**
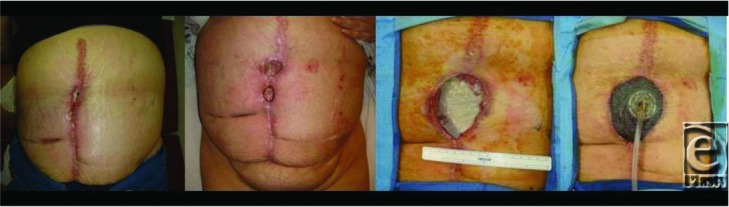


## DESCRIPTION

A 45-year-old woman presented for regular postoperative follow-up of an abdominal wall reconstruction with soft tissue dehiscence of her midline laparotomy wound. The underlying fascia was intact, with exposure of biologic mesh in the open wound. Negative pressure wound therapy (NPWT) was employed to promote healing of her wound.

## QUESTIONS

1. **What is NPWT?**
2. **What are the main advantages of NPWT and how does it work?**
3. **When is NPWT indicated for wound therapy?**
4. **What are some potential complications when using NPWT?**

## DISCUSSION

Negative pressure wound therapy, or NPWT, is a wound management technique that applies subatmospheric pressure to a wound to improve the healing process, oftentimes in the form of vacuum-assisted closure devices. Negative pressure wound therapy utilizes a foam dressing sealed with a semiocclusive film barrier, which is connected to a negative pressure pump; vacuum-assisted closure promotes granulation, wound contracture, and a decreased bacterial count by applying modulated negative pressure to both the dressing sponge and the wound.^1^ Rising comorbidities such as obesity and diabetes leave a significant population of patients with impaired wound healing. Subsequently, patients and the healthcare system spend considerable time and expense on basic wound maintenance (ie, twice daily dressing changes).^2^ Negative pressure wound therapy has gained momentum as a potential solution to many patients' wound management and healing needs. Studies show NPWT is a favorable option to heal chronic wounds for multiple reasons. The primary function is to decrease wound size and expedite time to healing,^2^ thereby decreasing risk of wound complications and skin dehiscence. In addition, NPWT also reduces the number of dressing changes prior to primary closure and is better tolerated by patients when compared with traditional wound dressings.^3^ These benefits ultimately lead to a decreased length of hospital stay with less morbidity and less interval procedures. Results from cost analysis studies report an overall decrease in costs using NPWT when compared with traditional wound therapy.^4^

Several hypotheses exist regarding the exact mechanisms of NPWT and how it accelerates wound healing. Negative pressure wound therapy works via 2 main modalities: mechanically through macrostrain and physiologically through microdeformation. Studies show that macrostrain (a) removes excess fluid (interstitial fluid with inflammatory mediators and/or infectious exudates that could impair wound healing), (b) helps maintain wound homeostasis, and (c) decreases tension on closed incisions.^9^ Microdeformation (a) improves perfusion, (b) increases angiogenesis through modifying gene expression and stimulating fibroblast migration,^6^^,^^7^ (c) increases cellular energy (ATP),^8^ (d) stimulates the formation of granulation tissue,^7^ and (e) promotes lymphangiogenesis.^10^

Negative pressure wound therapy is used in wounds that are refractory to traditional methods of management such as skin grafts and local/free flaps. Furthermore, some patients who are not candidates for traditional techniques or those with chronic wounds which lack healthy granulation tissue or optimal wound beds make excellent candidates for NPWT.^1^ Negative pressure wound therapy has been the mainstay in chronic wound therapy since its introduction to surgical wound management. It has proven useful for maintenance of open and uninfected wounds, notably diabetic ulcers.^3^ Current research shows NPWT has also been successfully used to help heal high-risk closed incisions,^5^ as well as open abdominal wounds,^11^ open chest wounds,^12^ pressure ulcers,^13^ skin grafts bolster, and traumatic injuries including burns and fractures.^14^ Recent studies have also advocated the use of instillation as a supplement to standard NPWT. During the instillation phase, the pump instills fluid into the wound. The instillation agent usually consists of an antibiotic irrigation fluid or a cleansing agent that is administered in between sessions of NPWT. Instillation cycles are typically set for 10 minutes in between every 3 hours of NPWT. This hybridized technology is generally reserved for complicated and contaminated wounds to facilitate removal of debris that may impede the healing process.^15^^,^^16^

Although rare, suboptimal outcomes related to NPWT have been documented. Often a product of wound location, the most significant potential complications are bleeding and infection directly related to the use of NPWT.^17^ Hence, caution should be used when using NPWT to avoid exposed organs or neurovasculature structures, to treat patients at risk for bleeding, and to manage deep wound sites, or heavily infected areas. Understanding of postsurgical anatomy of underlying structures is very important, and all retained material should be fully removed to prevent foreign body reactions and infection and/or gossypiboma.^18^ There is also an inherent challenge in the application of NPWT. Aseptic isolation of a complicated or irregular wound and a reliable seal for suction can be technically difficult to achieve. A common example of a challenging site for NPWT is a wound in close proximity to ostomies, which can increase the risk of contamination. Equally challenging are irregularly shaped wounds that cross skin folds and inherently moist areas of the body. Standardized protocol in vacuum maintenance should be followed at all times to optimize NPWT's effect on healing.

Negative pressure wound therapy is a well-developed technique utilizing simple concepts of negative pressure to enhance wound healing. It works via 2 main methods—macrostrain and microdeformation—that help to close wounds, maintain a clean environment, and promote healing at the cellular level. Negative pressure wound therapy is efficacious in a wide variety of patient populations and wound types. Although NPWT may not be indicated in every type of wound, it is an important technique that should be considered by all plastic surgeons encountering patients requiring wound care and maintenance.

**Figure:**